# Preoperative soluble VCAM‐1 contributes to predict late mortality after coronary artery surgery

**DOI:** 10.1002/clc.23443

**Published:** 2020-08-08

**Authors:** Ramon Corbalan, Mauricio Garcia, Luis Garrido‐Olivares, Lorena Garcia, Gonzalo Perez, Rosemarie Mellado, Ricardo Zalaquett, Mario Chiong, Jorge Quitral, Sergio Lavandero

**Affiliations:** ^1^ Division of Cardiovascular Diseases, Faculty of Medicine Pontificia Unversidad Catolica de Chile Santiago Chile; ^2^ Cardiovascular Surgery, Division of Surgery, Faculty of Medicine Pontificia Universidad Catolica de Chile Santiago Chile; ^3^ Advanced Center for Chronic Diseases (ACCDiS), Faculty of Chemical and Pharmaceutical Sciences and Faculty of Medicine Universidad de Chile Santiago Chile; ^4^ Faculty of Chemistry and Pharmacy Pontificia Unversidad Catolica de Chile Santiago Chile; ^5^ Corporacion Centro de Estudios Cientificos de las Enfermedades Cronicas (CECEC) Santiago Chile; ^6^ Cardiology Division University of Texas Southwestern Medical Center Dallas Texas USA

**Keywords:** atrial fibrillation, cardiovascular death, CHA2DS2‐VASc, soluble VCAM‐1

## Abstract

**Background:**

Soluble vascular cell adhesion molecule‐1 has been associated with long‐term cardiovascular mortality in patients with stable coronary artery disease and to the development of new atrial fibrillation in subjects with cardiovascular risk factors but no evidence of cardiac disease.

**Hypothesis:**

Preoperative soluble vascular cell adhesion molecule‐1 predicts the risk of future all‐cause death and cardiovascular death among patients submitted to elective coronary artery bypass surgery.

**Methods:**

From a cohort of 312 patients who underwent elective coronary artery bypass surgery prospectively followed for a median of 6.7 years, we evaluated the prognostic role of preoperative soluble vascular cell adhesion molecule‐1, inflammatory markers, CHA2DS2‐VASc score and development of postoperative atrial fibrillation (POAF). Univariable and multivariable Cox regression analyses were performed to establish an association of these parameters with long term all‐cause death and cardiovascular death.

**Results:**

During 2112 person‐years of follow‐up, we observed 41 deaths, 10 were cardiovascular deaths. Independently increased levels of preoperative soluble vascular cell adhesion molecule‐1, POAF, and CHA2DS2‐VASc score were associated with all‐cause mortality. After multivariate adjustment, elevated preoperative soluble vascular cell adhesion molecule‐1 and POAF were the only independent predictors of all‐cause death. Also, preoperative soluble vascular cell adhesion molecule‐1, POAF, and CHA2DS2‐VASc score resulted in being independent predictors of cardiovascular mortality.

**Conclusions:**

Increased circulating levels of preoperative soluble vascular cell adhesion molecule‐1, together with POAF and CHA2DS2‐VASc score, were significantly associated with future all‐cause death and cardiovascular death among patients submitted to coronary artery bypass surgery.

## 1 INTRODUCTION

VCAM‐1 is a membrane protein belonging to the superfamily of immunoglobulins and is mainly expressed in the endothelium. The primary role of VCAM‐1 is related to adhesion and transmigration of inflammatory cells (macrophages and dendritic cells) through the endothelium. Thus, VCAM‐1 is considered a biomarker for inflammation and endothelial activation.^1^

Soluble VCAM‐1 (sVCAM‐1) is generated by cleavage of the VCAM‐1 molecule by tumor necrosis factor‐alpha‐converting enzyme (TACE or ADAM 17) in response to cytokines. sVCAM‐1 has also been associated with all‐cause and cardiovascular mortality in patients with diabetes and advanced chronic kidney disease.^2^, ^3^

sVCAM‐1, a biomarker of endothelial dysfunction, has also been associated with the extension of atherosclerosis,^4^ long‐term cardiovascular mortality in patients with stable coronary artery disease and the development of new atrial fibrillation in subjects with cardiovascular risk factors but no evidence of baseline cardiac disease.^5^, ^6^, ^7^ Increased levels of sVCAM‐1 have been reported as predictors of postoperative atrial fibrillation (POAF) in patients undergoing on‐pump elective cardiac surgery.^8^, ^9^ This arrhythmia is linked to a higher risk of late adverse cardiac events in patients submitted to cardiac surgery.^10^, ^11^, ^12^ The prognostic role of CHA_2_DS_2−_VASc score that incorporates age and most cardiovascular risk factors is associated with a higher risk of POAF and stroke after cardiac surgery.^13^, ^14^, ^15^ However, the prognostic role of sVCAM‐1 in long term survival of patients submitted to cardiac surgery remains not understood.

The present study aimed to evaluate in a prospective cohort the relationship of sVCAM‐1, an inflammatory and endothelial dysfunction biomarker, together with other well known clinical predictors of adverse events such as POAF and CHA_2_DS_2_‐VASc score, to the risks of long term all‐cause mortality and cardiovascular death among patients submitted to elective on‐pump coronary artery bypass surgery.

## 2 METHODS

### 2.1 Patients

From April 2007 to January 2014, a prospective cohort of 300 and 12 patients, older than 18 years old and in normal sinus rhythm submitted to elective on‐pump cardiac surgery, was included in the study. Baseline clinical characteristics and laboratory tests, sVCAM‐1, high sensitivity C reactive protein (usCRP), leukocyte count, CHA_2_DS_2_‐VASc score, and two‐D echocardiograms were assessed before cardiac surgery. This study conforms to the Declaration of Helsinki and has been approved by our Ethics Committee at the school of medicine, Pontificia Unversidad Catolica de Chile. Written informed consent was obtained in all patients. This clinical study was registered in a public trials registry at FONDECYT 1141137 & 1 100 801 & 1 181 147 from the national agency for research development (ANID), Santiago, Chile).

The following exclusion criteria were applied: (a) nonelective cardiac surgery, (b) previous cardiac surgery, (c) combined valvular and coronary surgery, (d) rheumatic valve disease, (e) acute myocardial infarction (AMI) during the last 2 months, (f) cardiogenic shock at hospitalization, (g) history of malignancy, rheumatologic disease or any chronic inflammatory disease, (h) chronic steroidal treatment, (i) thyroid dysfunction, (j) evidence of active infection, and (k) history of paroxysmal or permanent AF.

### 2.2 Clinical, biochemical, and echocardiographic characteristics

The following preoperative variables were included: age, gender, associated comorbidities including hypertension, diabetes mellitus, and chronic obstructive pulmonary disease (COPD), previous cerebrovascular accident (CVA), heart failure, valvular heart disease, the severity of the coronary artery disease (Syntax score I), standard additive Euroscore, logistic Euroscore, CHA_2_DS_2_‐VASc score, and two‐D echocardiogram cardiac dimensions. Systolic left ventricular function was considered normal when left ventricular (LV) ejection fraction (EF) was higher than 50%, moderate to reduced when it was 40% to 50%, and decreased when EF was less than 40%. Clinical variables were defined using standard operational definitions.

POAF was defined as any new episode of AF lasting more than 15 minutes during the first 72 hours after surgery. Patients were monitored continuously with a telemetry system with automated arrhythmia detection (IntelliVue MP70, Phillips Healthcare, Andover, Massachusetts). In every patient with a suspected arrhythmic event, a standard 12‐lead Electrocardiogram (EKG) was performed and reviewed by a trained cardiologist. Management of POAF comprised oral anticoagulants and antiarrhythmic agents, which was recommended for 6 weeks; after that, this was left to treating physicians.

Biochemical variables: white blood cell (WBC), neutrophil count, and usCRP were utilized as inflammatory markers. WBC and neutrophil count were performed with an automated cell detector, and usCRP was evaluated using a high sensitivity immunoreagent (Dade Behring, Deerfield, Illinois). Soluble adhesion molecule VCAM (sVCAM‐1) was determined using a commercial A method to measure circulating biomarkers (Elisa) kit (R&D Systems Inc, Minneapolis, MM, USA).

Cardiac surgery: The surgical procedure consisted of an on‐pump aortocoronary bypass using a Terumo Sarns 8000 (Terumo Corporation, Tokyo, Japan) system and a standard cardiotomy suction setup. No aprotinin was used during or after surgery. Preoperative medications were collected as well as the following intraoperative variables: cardiopulmonary bypass time and aortic cross‐clamp time.

### 2.3 Primary and secondary outcomes

The primary outcome was all‐cause death in a time‐to‐event analysis. Secondary time‐to‐event outcome was cardiovascular death. This comprised: (1) cardiac death and (2) extracardiac cardiovascular death, which included stroke, aneurysmal dissections and ruptures, and pulmonary embolism. The causes of death are incorporated in the Chilean Civil Registry.

### 2.4 Statistical analysis

Descriptive statistics was performed for categorical and continuous variables. Categorical variables are reported as numbers and percentages. Normally distributed continuous variables are presented as mean ± SD (SD). Continuous variables with nonnormal distribution are presented as median (interquartile range [IQR]). Outcomes were death, cardiovascular death, and noncardiovascular death. Exploratory analysis between variables and outcomes was performed using Chi‐Square test. Univariate and multivariate Cox proportional hazard regression models were used to test the association between CHA_2_DS_2_‐VASc scores, POAF, sVCAM‐1, usCRP and neutrophil count, and the outcome all‐cause death. The proportional hazard assumption was evaluated using the Schoenfeld test in each model. Finally, independent Kaplan‐Meier curves were constructed using POAF, stratified CHA_2_DS_2_‐VASc scores, and stratified sVCAM‐1. CHA_2_DS_2_‐VASc score was stratified in three groups, 0 to 1 points, 2 to 4 points, and greater or equal to 5 points. sVCAM‐1 values were stratified in tertiles (< 629, 629‐962 and > 962 ng/mL. As a sensitivity analysis, we also stratified sVCAM‐1 using percentile 50 (777) and the empirical optimal cutpoint using Liu criteria (989). Log‐rank test was also employed to test differences in the probability of cardiovascular death between population strata. No sensitivity analysis for missing data was performed. All statistical analyses were performed with the STATA SE 15.1 (StataCorp LP, College Station, Texas). All p values were based on two‐sided test and were considered statistically significant at *P* < .05. Forest plot figures were generated in R, version 3.6.1, using RStudio version 1.1.456.

## 3 RESULTS

### 3.1 Study cohort and follow‐up

There were 312 patients who fulfilled the inclusion criteria of this study. The median follow‐up was 6.7 (5.5‐8.7) years. Follow‐up finished by December 2016 and was completed in all patients.

### 3.2 Baseline clinical and surgical data

Baseline demographics and clinical characteristics of the study participants are shown in Table 1. The mean age was 64 ± 10 years, and 85.3% (266) were male. Coronary surgery was performed using the left internal mammary artery in 283 patients (90.7%). Cardiopulmonary bypass time was 97 (79‐117) minutes and aortic cross‐clamp time 61 (47‐77) minutes. Most patients (76%) had a normal left ventricular function.

**TABLE 1:** Baseline patient characteristics and preoperative medications

	Patients (n = 312) (%)
Demographics	
Age, y	64 ± 10
Female	46 (15)
Medical history	
Hypertension	229 (73)
Diabetes mellitus	113 (36)
COPD	7 (2)
CVA	9 (3)
Heart failure	35 (11)
Valvular heart disease	3 (1)
Biochemical and hematological tests	
Creatinine (mg/dL)	1.0 ± 0.5
Potassium (mEq/L)	4.2 ± 0.7
White blood cell count (10^3^/mL)	7.554 ± 1.860
usCRP (mg/dL)	0.8 (0.2‐3.2)
Neutrophil count (10^3^/mL)	4.928 ± 3.663
Hemoglobin (g/L)	14 (3)
TSH (mUI/L)	2.8 (1.1‐3.3)
Cardiac parameters	
Normal LV function	236 (76)
Moderate LV dysfunction	67 (21)
Severe LV dysfunction	9 (3)
Left atrial diameter (mm)	33 ± 17
Euroscore	
Additive	3 (2‐5)
Logistic	0.23 (0.17‐0.42)
Coronary disease	
Syntax score I	20 (14‐25)
Baseline medications	
Statins	212 (72)
ACE inhibitors	160 (55)
Spironolactone	12 (4)
Beta‐blockers	195 (66)
Antiarryhthmics	0 (0)

*Note*: Values expressed as mean ± SD or n (%) or percentile (pc25‐75).

Abbreviations: CVA: cerebrovascular accident, COPD: Chronic obstructive pulmonary disease, LV: left ventricular, TSH: Thyroid stimulating hormone, usCRP: highly sensitivity C reactive protein.

### 3.3 POAF, CHA_2_DS_2_‐Vasc score, and sVCAM‐1

**FIGURE 1:**
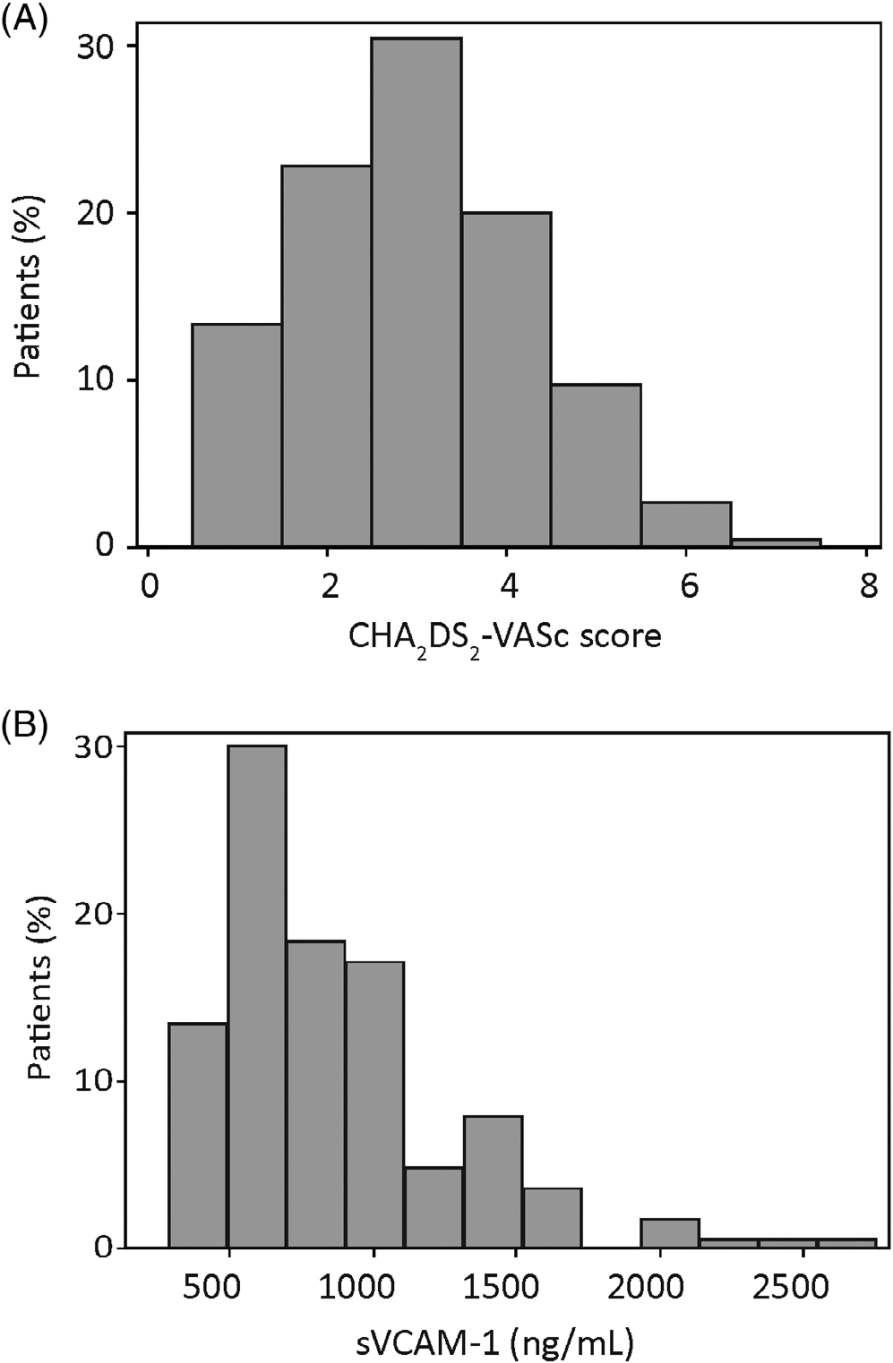
A, Histogram of CHA_2_DS_2_‐VASc scores. B, Histogram of preoperative sVCAM‐1

POAF was observed in 53 patients (17%). Median CHA_2_DS_2_‐VASc score was three,^2^, ^3^, ^4^ and the histogram is presented in Figure 1A. Preoperative sVCAM‐1 was available in 162 patients (51.9%). Median sVCAM‐1 was 777 (587‐1049) ng/mL, and the histogram is presented in Figure 1B.

### 3.4 All‐cause death and cardiovascular death

The primary outcome all‐cause death occurred in 42 patients (13.5%) in 2112 person‐years of follow‐up. The secondary outcome, cardiovascular death was present in 22 patients (52.4% of all death and 7% of the cohort). Among them, five patients (1.6%) had a lethal myocardial infarction, and two patients (0.6%) died due to ischemic stroke (Table S1). Noncardiovascular death occurred in 20 patients (47.6%).

### 3.5 All‐cause death

Hazard ratios (HR) and confidence interval (CI) corresponding to univariable Cox proportional hazard regressions are reported in Table S2. POAF (HR: 3.95; CI: 2.12‐7.36; *P* < .001), CHA_2_DS_2_‐VASc scores (HR: 1.66 for each incremental point; CI: 1.33‐2.06; *P* < .001) and sVCAM‐1 (HR: 1.002 for each incremental ng/mL; CI: 1.001‐1.002; *P* < .001) were statistically significant variables associated to all‐cause death. Besides, age, diabetes mellitus, valvular heart disease, congestive heart failure, and additive Euroscore were statistically significant variables associated with all‐cause death.

**FIGURE 2:**
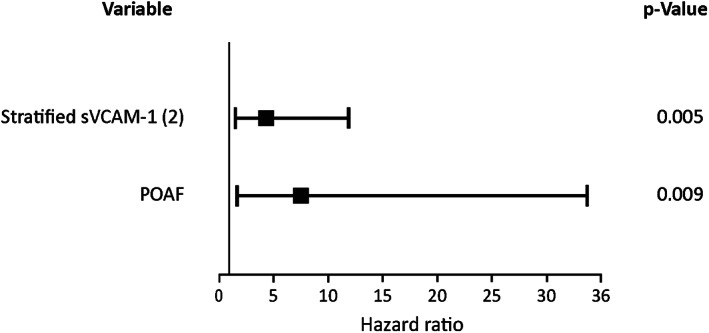
Forest plot of hazard ratio and 95% confidence interval (sVCAM‐1 stratified using percentile 50)

After multivariable adjustment elevated sVCAM‐1 (HR: 1.0012 for each ng/mL; CI: 1.0005‐1.0019; *P* = .001) and POAF (HR: 4.14; CI: 1.4‐11.8, *P* = .008) were the only independent predictors of all‐cause death in this population (Table S3). The Forest plot of HR and 95% CI, corresponding to the multivariate Cox proportional hazard model is shown in Figure 2.

**FIGURE 3:**
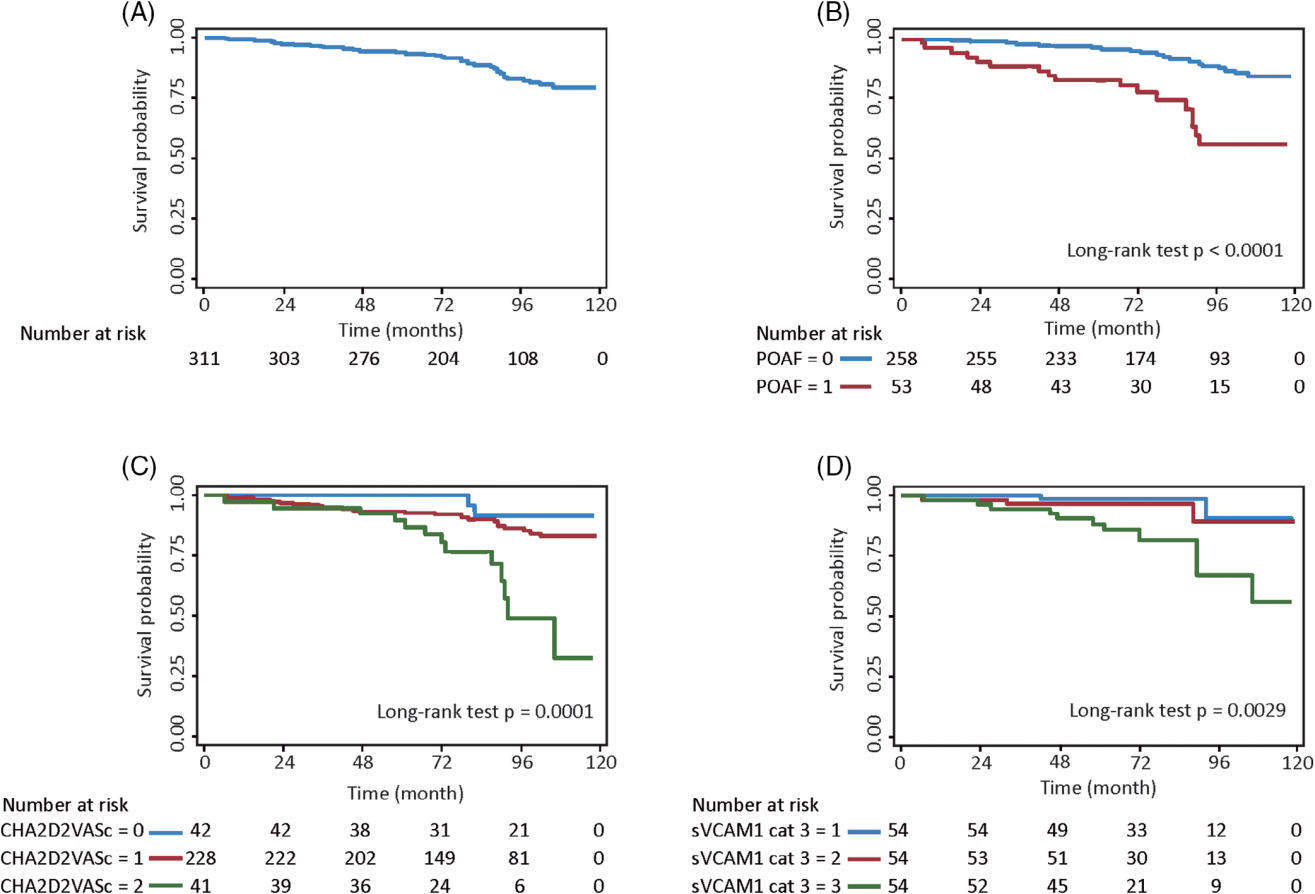
Kaplan–Meier curves of all‐cause death A, Unstratified cohort, B, stratified by POAF, C, stratified by CHA_2_DS_2_‐VASc scores, and D, stratified sVCAM‐1 (tertiles)

Kaplan‐Meier curves of all‐cause death for POAF, stratified CHA_2_DS_2_‐VASc scores, and stratified sVCAM‐1 are shown in Figure 3A‐D. Kaplan‐Meier curves for alternative stratifications of sVCAM‐1 are also shown in Figures [Link], [Link].

Also, diabetes mellitus, cerebrovascular accident, valvular heart disease, and congestive heart failure were statistically significant variables associated with all‐cause death.

### 3.6 Cardiovascular death

HR and CI corresponding to univariable Cox proportional hazard regressions are reported in Table S3. POAF (HR: 3.41; CI: 1.41‐8.23; *P* = .006), CHA_2_DS_2_‐VASc scores (HR: 1.76 for each incremental point; CI: 1.30‐2.39; *P* < .001) and sVCAM‐1 (HR: 1.002 for each incremental ng/mL; CI: 1.001‐1.002; *P* < .001) were statistically significant variables associated to cardiovascular death.

**FIGURE 4:**
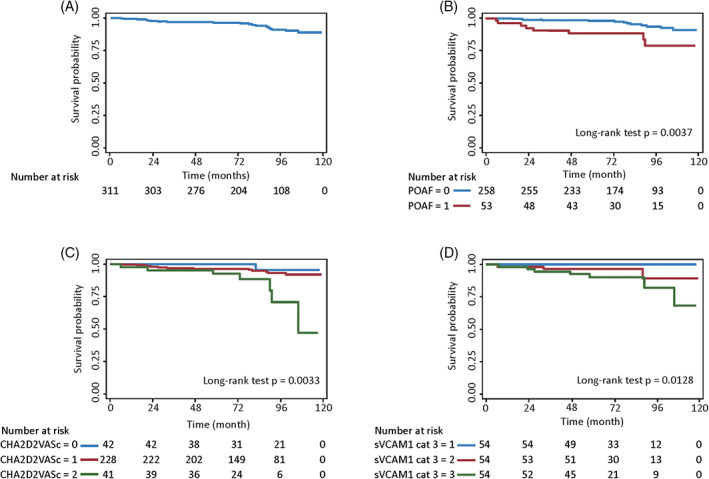
Kaplan–Meier curves of cardiovascular death A, Un‐stratified cohort, B, stratified by POAF, C, stratified by CHA_2_DS_2_‐VASc scores, and D, stratified sVCAM‐1 (tertiles). POAF, postoperative atrial fibrillation

Kaplan‐Meier curves of all‐cause death for POAF, stratified CHA_2_DS_2_‐VASc scores, and stratified sVCAM‐1 are shown in Figure 4A‐D. Interestingly, sVCAM‐1 levels did not correlate with the Syntax score.

## 4 DISCUSSION

### 4.1 Main findings

The present work shows that endothelial dysfunction, assessed by preoperative sVCAM‐1 levels, is associated with late all‐cause mortality and cardiovascular death in patients with coronary artery disease submitted to elective on‐pump coronary artery bypass surgery. These findings are independent of inflammatory markers and other cardiovascular risk factors.

### 4.2 Comparison with published data

Endothelial dysfunction has been associated with adverse cardiovascular events in patients with coronary artery disease. Blankenberg et al evaluated the prognostic role of multiple biomarkers in a large prospective study of patients with stable coronary artery disease and found that sVCAM‐1 was a significant predictor of future death in these patients.^6^ Peter et al have previously shown that sVCAM‐1 correlates with the extent of atherosclerosis in patients with peripheral artery disease.^5^ More recently, Mu et al reported that the expression of sVCAM‐1 was increased in aortic tissues of atherosclerotic patients.^16^ These findings lead a group of investigators to speculate about the relationship of soluble adhesion molecule‐1 with cardiovascular mortality.^17^ sVCAM‐1 has been reported as a predictor of late cardiovascular death in patients with diabetes mellitus type 2 and hemodialysis.^2^, ^3^ Also, sVCAM‐1 has also been reported to be an independent predictor of all‐cause mortality in sepsis and critical illness.^18^, ^19^

The expression of endocardial VCAM‐1 increases up to double in patients with AF as well as after rapid atrial stimulation.^20^ In a previous independent study, we evaluated a prospective cohort of 144 patients submitted to elective cardiac surgery with clinical and echocardiographic variables and biomarkers of inflammation, oxidative stress, and endothelial dysfunction. We found that sVCAM‐1 was the only significant predictor of POAF.^8^ These findings have been corroborated by Harling et al.^9^ The Bruneck Study evaluated a large variety of biomarkers in a cohort of patients with cardiovascular risk factors, and only sVCAM‐1 resulted in an independent predictor of new AF.^7^

The present study shows for the first time that sVCAM‐1 is a predictor for late all‐cause death and cardiovascular mortality in patients submitted to elective on‐pump coronary artery bypass surgery. Given that this factor can be measured before cardiac surgery, this marker allows to identify patients with a higher risk of cardiovascular death and eventually optimize preventive, therapeutic measures in both observational and interventional studies.

The adverse prognostic of POAF has already been reported in the long‐term follow up after cardiac surgery,^7^, ^8^, ^9^, ^10^, ^21^, ^22^, ^23^ and this has also been confirmed in the present study. Initially, this arrhythmia was considered benign and associated with prolonged hospitalization and higher health costs, but large series reported an association with higher risks of stroke and late cardiovascular mortality.^10^

In a previous study of a retrospective cohort of 2385 patients submitted to cardiac surgery, Kashani et al reported an increased risk of developing POAF with an HR: 1.27 (CI: 1.18‐1.36) for every point of the CHA_2_DS_2_‐VASc score.^14^ Likewise, Biancari et al evaluated 1226 patients free of previous AF and subjected to cardiac surgery. They observed that a higher CHA_2_DS_2_‐VASc score was associated with an elevated risk of ischemic stroke and late mortality.^15^ Similar findings have also been reported by Peguero et al.^24^ In agreement with these previous studies, our work confirmed that CHA_2_DS_2_‐VASc score is a significant predictor of late mortality after cardiac surgery.

The present findings show that sVCAM‐1, a biomarker of endothelial dysfunction, is an independent predictor of late all‐cause mortality and cardiovascular death after coronary artery bypass surgery. Also, POAF, and CHA_2_DS_2_‐VASc score add to an adverse prognosis in these patients.

### 4.3 Limitations and future investigations

Our study has several limitations. First, its relatively small sample size and consequently limited power may have obscured other subtle associations. Second, the incomplete assessment of endothelial function may have limited the estimation of HR presented here. Third, the population evaluated was a heterogeneous sample of patients with stable coronary artery disease, a few of them with heart failure, which may affect their long term prognosis. Finally, we only performed perioperative measurements without assessing the subsequent impact of the surgical act and medical therapy in the levels of sVCAM‐1 during the follow‐up period.

Future studies should investigate the prognostic role of sVCAM‐1 in separate cohorts of patients with stable coronary artery disease submitted to elective cardiac surgery.

## 5 CONCLUSIONS

In summary, this is the first study to identify an association between increased levels of sVCAM‐1 and future death and cardiovascular death among patients submitted to elective on‐pump coronary artery bypass surgery.

## CONFLICT OF INTEREST

The authors declare no potential conflict of interests.

## Supporting information


**TABLE S1** Follow‐up, CHA2DS2‐VASc score and outcomes
**TABLE S2:** Cox proportional hazard regression models (outcome total death)
**TABLE S3:** Univariable Cox proportional hazard regression models (outcome CV death)

**FIGURE S1** Kaplan‐Meier curves of all‐cause death according to stratified sVCAM‐1 (cutpoint using percentile 50 [777 ng/mL]).

**FIGURE S2** Kaplan‐Meier curves of all‐cause death according to stratified sVCAM‐1 (cutpoint using Liu criteria [989 ng/mL]).

**FIGURE S3** Kaplan‐Meier curves of cardiovascular death according to stratified sVCAM‐1 (cutpoint using percentile 50 [777 ng/mL]).

**FIGURE S4** Kaplan‐Meier curves of cardiovascular death according to stratified sVCAM‐1 (cutpoint using Liu criteria [989 ng/mL]).

## References

[1] Pepinsky B , Hession C , Chen LL , et al. Structure/function studies on vascular cell adhesion molecule‐1. J Biol Chem. 1992;267:17820‐17826. 1381355

[2] de Jager J , Dekker JM , Kooy A , et al. Endothelial dysfunction and low‐grade inflammation explain much of the excess cardiovascular mortality in individuals with type 2 diabetes. Hoorn Study Arterioescl Thromb Vasc Biol. 2006;26:1086‐1093. 10.1161/01.ATV.0000215951.36219.a4 16514084

[3] Chiang JF , Han SP , Pai MF , et al. High soluble vascular cell adhesion molecule‐1concentrations predict long‐term mortality in hemodyalisis patients. Int Urol Nephrol. 2013;45:1693‐1701. 23563803 10.1007/s11255-013-0425-z

[4] Cybulsky MI , Iiyama K , Li H , et al. A major role for VCAM‐1, but not ICAM‐1, in early atherosclerosis. J Clin Invest. 2001;107:1255‐1262. 11375415 10.1172/JCI11871 PMC209298

[5] Peter K , Nawroth P , Conradt C , et al. Circulating vascular cell adhesion molecule‐1 correlates with the extent of human atherosclerosis in contrast to circulating intercellular adhesion molecule‐1, E‐selectin, P‐selectin, and thrombomodulin. Arterioscler Thromb Vasc Biol. 1997;17:505‐512. 9102169 10.1161/01.atv.17.3.505

[6] Blankenberg S , Rupprecht HJ , Bickel C , et al. Circulating cell adhesion molecules and death in patients with coronary artery disease. Circulation. 2001;104:1336‐1342. 11560847 10.1161/hc3701.095949

[7] Willeit K , Pechlaner R , Willeit P , et al. Association between vascular cell adhesion molecule 1 and atrial fibrillation. JAMA Cardiol. 2017;2:516‐523. 28355442 10.1001/jamacardio.2017.0064 PMC5814989

[8] Verdejo H , Roldan J , Garcia L , et al. Systemic vascular cell adhesion molecule‐1 predicts the occurrence of post‐operative atrial fibrillation. Int J Cardiol. 2011;150:270‐276. 20447702 10.1016/j.ijcard.2010.04.033

[9] Harling L , Lambert J , Ashrafian H , Darzi A , Gooderham NJ , Athanasiou T . Pre‐operative serum VCAM‐1 as a biomarker of atrial fibrillation after coronary artery bypass grafting. J Cardiothorac Surg. 2017;12:70. 28821262 10.1186/s13019-017-0632-2 PMC5563046

[10] Al‐Shaar L , Schwann TA , Kabour A , Habib RH . Increased late mortality after coronary artery bypass surgery complicated by isolated new‐onset atrial fibrillation: a comprehensive propensity‐matched analysis. J Thorac Cardiovasc Surg. 2014;148:1860‐1868. 24928262 10.1016/j.jtcvs.2014.05.020

[11] El‐Chami MF , Kilgo P , Thourani V , et al. New‐onset atrial fibrillation predicts long‐term mortality after coronary artery bypass graft. J Am Coll Cardiol. 2010;55:1370‐1376. 20338499 10.1016/j.jacc.2009.10.058

[12] Greenberg JW , Lancaster TS , Schuessler RB , Melby SJ . Postoperative atrial fibrillation following cardiac surgery: a persistent complication. Eur J Cardiothorac Surg. 2017;52:665‐672. 28369234 10.1093/ejcts/ezx039

[13] Chua SK , Shyu KG , Lu MJ , et al. Clinical utility of CHADS2 and CHA2DS2‐VASc scoring systems for predicting postoperative atrial fibrillation after cardiac surgery. J Thorac Cardiovasc Surg. 2013;146:919‐926. 23628495 10.1016/j.jtcvs.2013.03.040

[14] Kashani RG , Sareh S , Genovese B , et al. Predicting postoperative atrial fibrillation using CHA2DS2‐VASc scores. J Surg Res. 2015;198:267‐272. 26004496 10.1016/j.jss.2015.04.047

[15] Biancari F , Mahar MA , Kangasniemi OP . CHADS2 and CHA2DS2‐VASc scores for prediction of immediate and late stroke after coronary artery bypass graft surgery. J Stroke Cerebrovasc Dis. 2013;22:1304‐1311. 23253529 10.1016/j.jstrokecerebrovasdis.2012.11.004

[16] Mu W , Gong Z , Zheng F , Xing Q . Expression of vascular cell adhesion molecule‐1 in the aortic tissues of atherosclerotic patients and the associated clinical implications. Exp Ther Med. 2015;10:423‐428. 26622332 10.3892/etm.2015.2540 PMC4509110

[17] Becker A , van Hinshberg VW , Kostense PJ , et al. Is soluble intercelular adhesión molecule 1 related to cardiovascular mortality? Eur J Clin Invest. 2002;32:1‐8. 10.1046/j.1365-2362.2002.00919.x 11851720

[18] Skibstead S , Jones AE , Puscarich MA , et al. Biomarkers of endotelial activation in early sepsis. Shock. 2013;39:427‐432. 23524845 10.1097/SHK.0b013e3182903f0d PMC3670087

[19] Mikacenic C , Hahn WO , Price BL , et al. Biomarkers of endothelial activation are associated with poor outcome in critical illness. PLoS One. 2015;10:e0141251. 26492036 10.1371/journal.pone.0141251 PMC4619633

[20] Goette A , Bukowska A , Lendeckel U , et al. Angiotensin II receptor blockade reduces tachycardia‐induced atrial adhesion molecule expression. Circulation. 2008;117:732‐742. 18227384 10.1161/CIRCULATIONAHA.107.730101

[21] Shen J , Lall S , Zheng V , Buckley P , Damiano RJ , Schuessler RB . The persistent problem of new‐onset postoperative atrial fibrillation: a single institution experience over two decades. J Thorac Cardiovasc Surg. 2011;141:559‐570. 20434173 10.1016/j.jtcvs.2010.03.011 PMC2917532

[22] Villareal RP , Hariharan R , Liu B , et al. Postoperative atrial fibrillation and mortality after coronary artery bypass surgery. J Am Coll Cardiol. 2004;43:742‐748. 14998610 10.1016/j.jacc.2003.11.023

[23] Attaran S , Shaw M , Bond L , Pulla M , Fabri B . Atrial fibrillation postcardiac surgery: a common but a morbid complication. Interact Cardiovasc Thorac Surg. 2011;12:772‐777. 21357310 10.1510/icvts.2010.243782

[24] Peguero JG , Issa O , Podesta C , Elmahdy HM , Santana O , Lamas GA . Usefulness of the CHA2DS2VASc score to predict postoperative stroke in patients having cardiac surgery independent of atrial fibrillation. Am J Cardiol. 2015;115:758‐762. 25616533 10.1016/j.amjcard.2014.12.037

