# Supplementary material for: Preoperative soluble VCAM‐1 contributes to predict late mortality after coronary artery surgery
Source: Clin Cardiol. 2020 Aug 8;43(11):1301–7. doi: 10.1002/clc.23443 (PMC7661653; doi:10.1002/clc.23443)
Supplement: Supplementary file 1 — TABLE S1 Follow‐up, CHA2DS2‐VASc score and outcomes TABLE S2: Cox proportional hazard regression models (outcome total death) TABLE S3: Univariable Cox proportional hazard regression models (outcome CV death) [file CLC-43-1301-s001.docx]

| **Table S1:** Follow-up, CHA2DS2-VASc score and outcomes | |
| --- | --- |
|  | Patients  (n=312) (%) |
| Follow-up (months) | 80 [66-105] |
| CHA2DS2-VASc score | 3 [2-4] |
| POAF | 53 (17) |
| ***Outcomes*** |  |
| Total death | 42 (13.5) |
| CV death | 22 (7) |
| Lethal AMI | 5 (2) |
| Lethal CVA | 2 (1) |
| Non-CV death | 20 (6.4) |
| Death at 30 days | 10 (3.2) |
| POAF or CV death | 67 (21.5) |
| POAF or total death | 78 |

Values expressed as n (%) or percentile [pc 25-75]. AMI: acute myocardial infarction, CVA: cerebrovascular accident, CV: cardiovascular, POAF: post‑operative atrial fibrillation.

**Table S2:** Cox proportional hazard regression models (outcome total death)

|  | |  | |  | |  | |
| --- | --- | --- | --- | --- | --- | --- | --- |
| Univariable models | |  | |  | |  | |
|  | | HR | | CI | | *p* | |
| ***Demographics*** |  | |  | |  | | |
| Female | | 1.72 | | 0.79-3.75 | | | 0.169 |
| Age | | 1.06 | | 1.02-1.10 | | | 0.001 |
| ***Medical history*** | |  | |  | | |  |
| Hypertension | | 1.50 | | 0.69-3.25 | | | 0.304 |
| Diabetes mellitus | | 2.45 | | 1.32-4.53 | | | 0.004 |
| COPD | | 1.86 | | 0.45-7.72 | | | 0.392 |
| CVA | | 2.13 | | 0.51-8.82 | | | 0.30 |
| Valvular heart disease | | 7.74 | | 1.84-32.54 | | | 0.005 |
| Congestive heart disease | | 2.46 | | 1.17-5.15 | | | 0.017 |
| ***Variable*** | |  | |  | | |  |
| CHA_2_DS_2_-VASc score | | 1.66 | | 1.33-2.06 | | | <0.001 |
| POAF | | 3.95 | | 2.12-7.36 | | | <0.001 |
| sVCAM-1 | | 1.002 | | 1.001-1.002 | | | <0.001 |
| usCRP | | 1.006 | | 0.98-1.02 | | | 0.518 |
| Neutrophil count | | 0.99 | | 0.88-1.11 | | | 0.90 |
| Syntax score I | | 1.03 | | 0.99-1.07 | | | 0.075 |
| Additive Euroscore | | 1.33 | | 1.12-1.58 | | | 0.001 |
|  | |  | |  | | |  |
| Multivariable model * | |  | |  | | |  |
|  | | HR | | CI | | | *p* |
| sVCAM-1 | | 1.0012 | | 1.0005 -1.0019 | | | 0.001 |
| POAF | | 4.14 | | 1.4‑11.8 | | | 0.008 |

CI: Confidence interval, CVA: cerebrovascular accident, COPD: Chronic obstructive pulmonary disease, HR: Hazard ratio, POAF: Post-operative atrial fibrillation, sVCAM‑1: Soluble vascular cell adhesion molecule-1, usCRP: high sensitivity C reactive protein.

* n=162. Harrel’s C = 0.83

**Table S3:** Univariable Cox proportional hazard regression models (outcome CV death)

|  | | HR | | | CI | | | *p* |
| --- | --- | --- | --- | --- | --- | --- | --- | --- |
| ***Demographics*** |  | | |  | | |  | |
| Female | | | 2.28 | | | 0.83-6.28 | | 0.110 |
| Age | | | 1.03 | | | 0.99-1.08 | | 0.148 |
| ***Medical history*** | | |  | | |  | |  |
| Hypertension | | | 1.55 | | | 0.52-4.61 | | 0.429 |
| Diabetes mellitus | | | 2.29 | | | 0.97-5.41 | | 0.058 |
| COPD | | |  | | |  | | 1.0 |
| CVA | | | 4.28 | | | 0.99-18.4 | | 0.051 |
| Valvular heart disease | | | 7.61 | | | 0.99-58.0 | | 0.05 |
| Congestive heart disease | | | 3.51 | | | 1.36-9.06 | | 0.009 |
| ***Variables*** | | |  | | |  | |  |
| CHA_2_DS_2_-VASc score | | | 1.76 | | | 1.30-2.39 | | <0.001 |
| POAF | | | 3.41 | | | 1.41-8.23 | | 0.006 |
| sVCAM-1 | | | 1.002 | | | 1.001-1.002 | | <0.001 |
| usCRP | | | 1.008 | | | 0.98-1.03 | | 0.42 |
| Neutrophil count | | | 0.76 | | | 0.57-1.01 | | 0.06 |
| Syntax Score I | | | 0.98 | | | 0.93-1.04 | | 0.603 |
| Additive Euroscore | | | 1.18 | | | 0.93-1.50 | | 0.189 |

CVA: cerebrovascular accident, CI: Confidence interval, COPD: Chronic obstructive pulmonary disease, HR: Hazard ratio, POAF: Post-operative atrial fibrillation, sVCAM-1: Soluble vascular cell adhesion molecule-1, usCRP: high sensitivity C reactive protein.
