# Supplementary figures and images for: Preoperative soluble VCAM‐1 contributes to predict late mortality after coronary artery surgery
Source: Clin Cardiol. 2020 Aug 8;43(11):1301–7. doi: 10.1002/clc.23443 (PMC7661653; doi:10.1002/clc.23443)

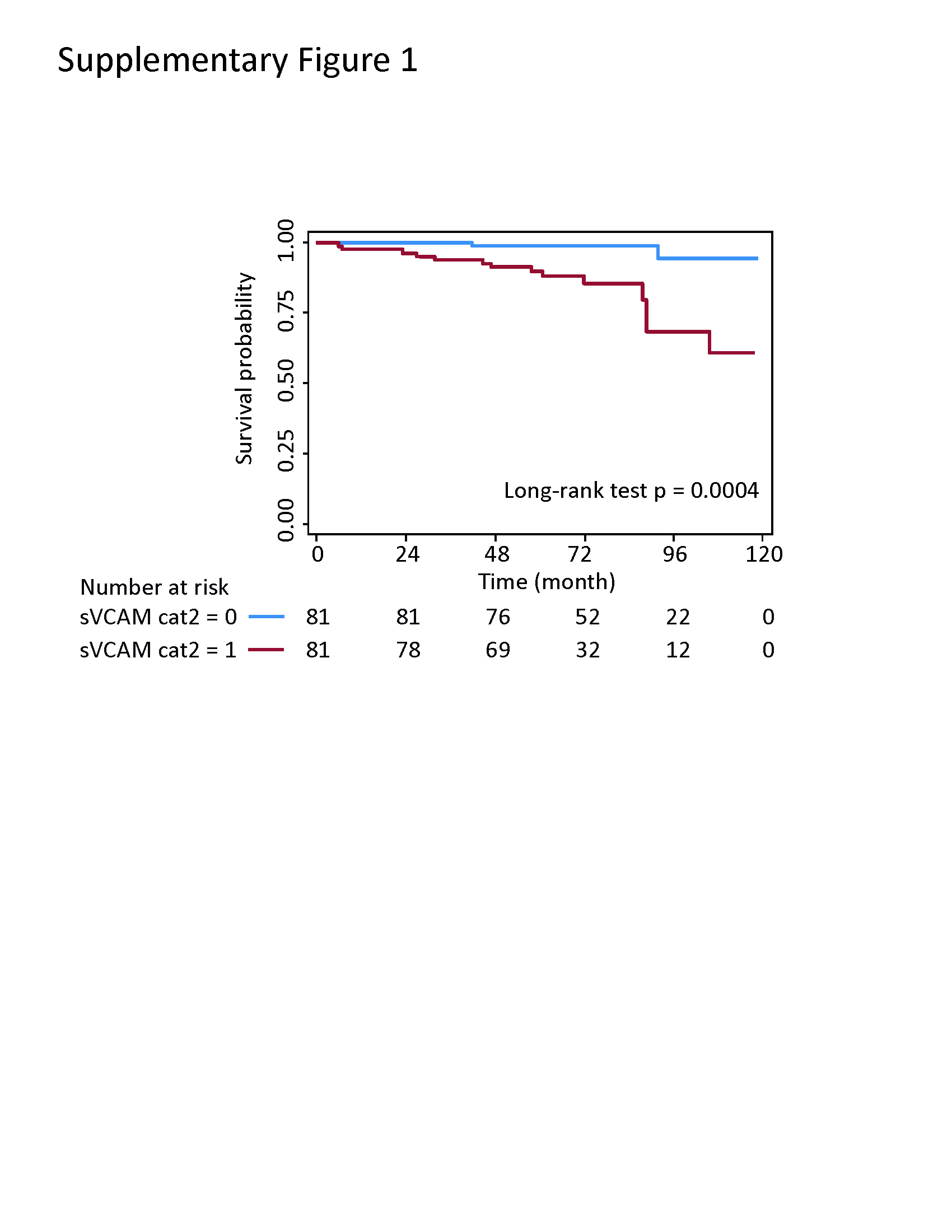

Supplement: Supplementary file 2 — FIGURE S1 Kaplan‐Meier curves of all‐cause death according to stratified sVCAM‐1 (cutpoint using percentile 50 [777 ng/mL]). [file CLC-43-1301-s002.tif]

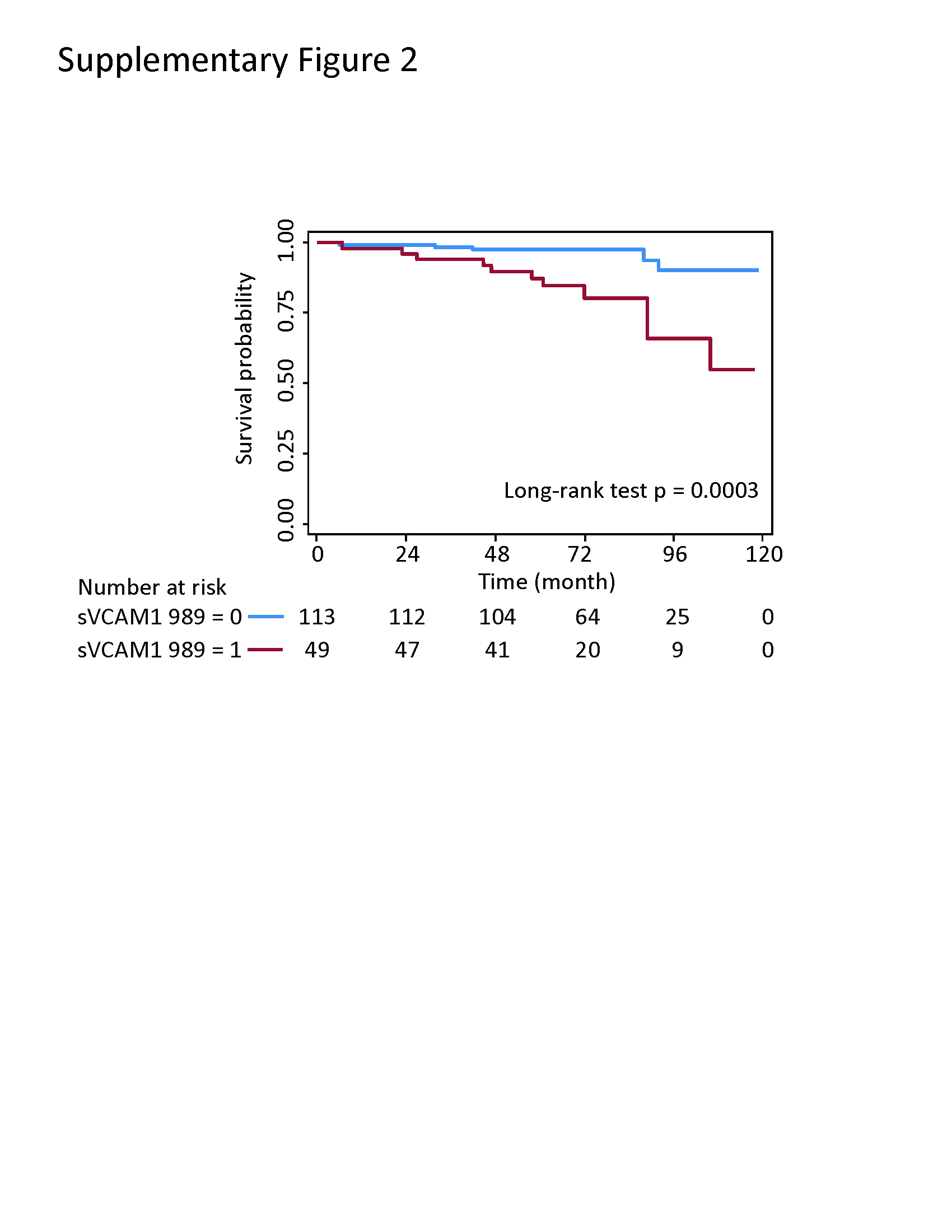

Supplement: Supplementary file 3 — FIGURE S2 Kaplan‐Meier curves of all‐cause death according to stratified sVCAM‐1 (cutpoint using Liu criteria [989 ng/mL]). [file CLC-43-1301-s003.tif]

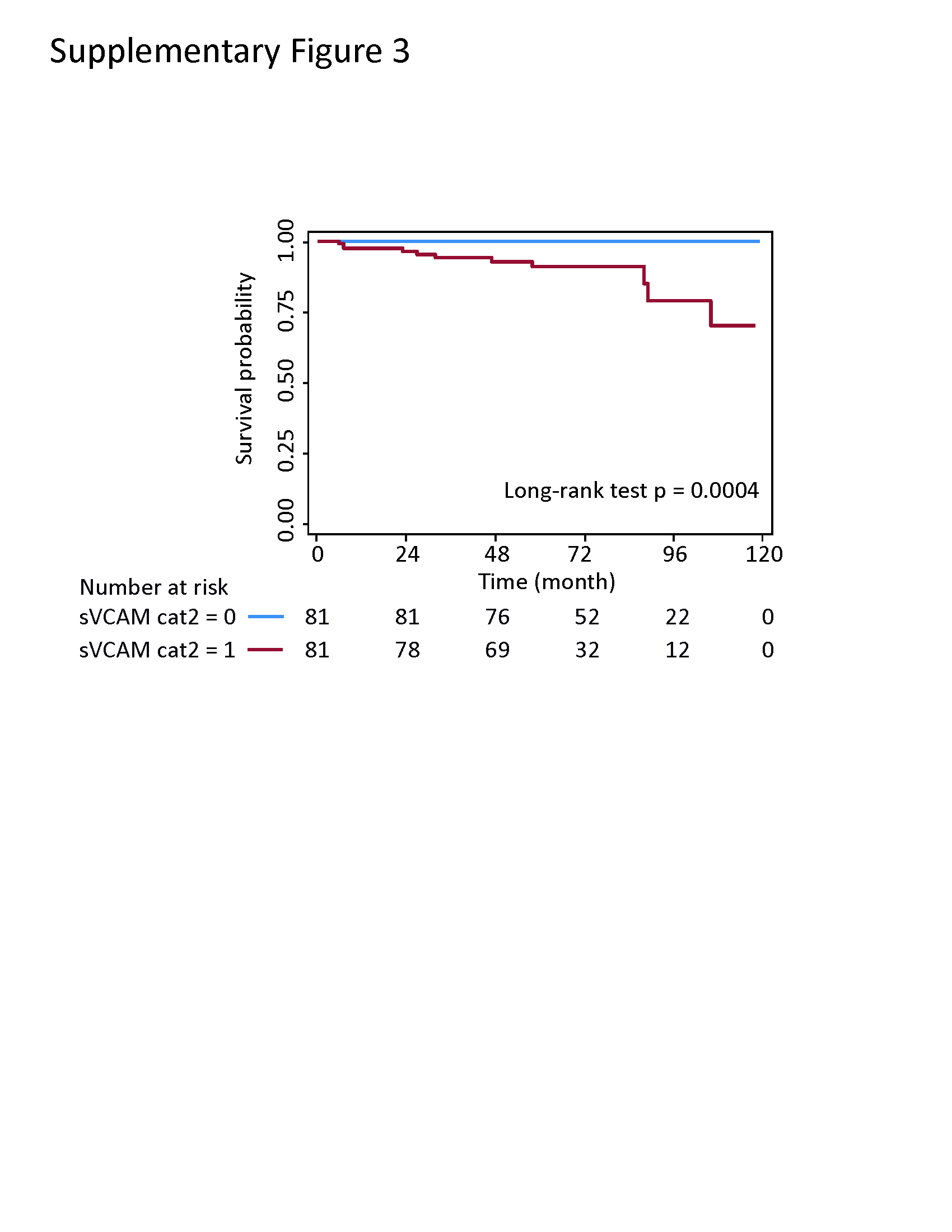

Supplement: Supplementary file 4 — FIGURE S3 Kaplan‐Meier curves of cardiovascular death according to stratified sVCAM‐1 (cutpoint using percentile 50 [777 ng/mL]). [file CLC-43-1301-s004.tif]

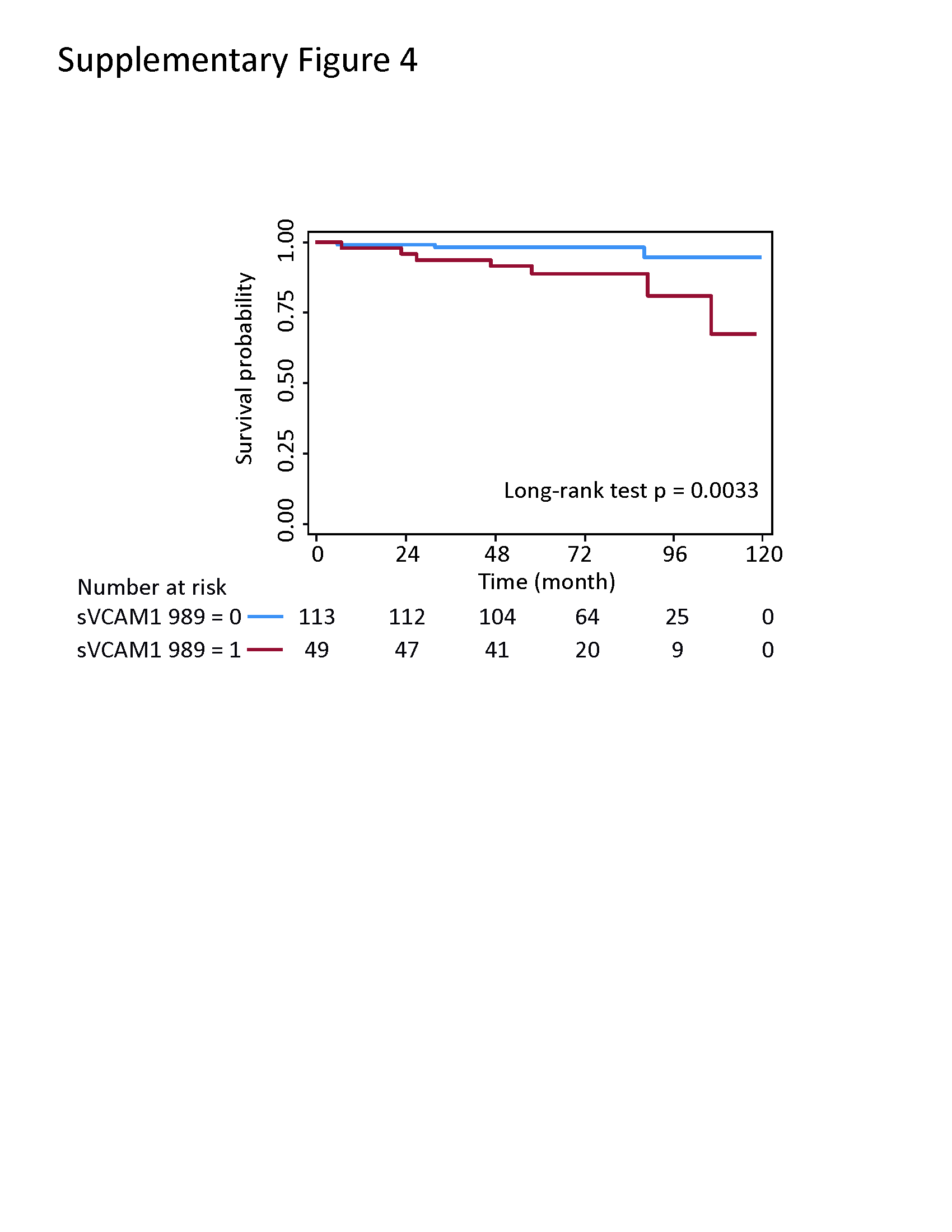

Supplement: Supplementary file 5 — FIGURE S4 Kaplan‐Meier curves of cardiovascular death according to stratified sVCAM‐1 (cutpoint using Liu criteria [989 ng/mL]). [file CLC-43-1301-s005.tif]
